# Supplementary material for: The Allosteric Activation of α7 nAChR by α-Conotoxin MrIC Is Modified by Mutations at the Vestibular Site
Source: Toxins (Basel). 2021 Aug 10;13(8):555. doi: 10.3390/toxins13080555 (PMC8402416; doi:10.3390/toxins13080555)
Supplement: Supplementary file 1 [file toxins-13-00555-s001.zip › toxins-1258977 supplementary 3.pdf]

Supplementary Materials: The Allosteric Activation of α7 nAChR by α-conotoxin MrIC is Modified by Mutations at the Vestibular Site

Alican Gulsevin, Roger L. Papke, Clare Stokes, Hue N. T. Tran, Aihua H. Jin, Aihua H Jin and Jens Meiler

Table S1. The average scores and standard deviations (S.D.) calculated for the ten lowest-scoring poses from the WT, G83V, A102V, and T106A peptide docking calculations. All units are in Rosetta Energy Units (REU).

Protein	Average Score (REU)	S.D. (REU)
WT	-25.9	0.8
G83V	-28.7	0.8
A102V	-28.0	0.6
T106A	-26.8	1.0

Table S2. The Shapiro-Wilk test statistic values and the p-values calculated for the different MrIC, GAT107, and PNU-120596 applications at different mutations to test whether the measurements fall within normal distribution. A p-value > 0.05 indicates a normal distribution for the set of measurements and a p-value < 0.05 indicates a non-normal distribution for the set of measurements.

Application	Shapiro Test Statistic	Shapiro p-value
WT MrIC + PNU	0.599909306	0.0004576
WT MrIC + PNU + TMP-TQS	0.859702408	0.227181956
α7C190A GAT107	0.963213861	0.845787644
α7C190A GAT107 + TMP-TQS	0.95107913	0.739512026
α7C190A MrIC + PNU	0.945044458	0.700046778
α7C190A MrIC + PNU + TMP-TQS	0.805926859	0.046861239
α7G83V MrIC + PNU	0.871676564	0.304304093
α7G83V PNU	0.94775176	0.709198117
α7A102V MrIC + PNU	0.669861794	0.002928978
α7A102V PNU	0.802413106	0.043275245
α7T106A MrIC + PNU	0.625163436	0.000912655
α7T106A PNU	0.633596778	0.000668759

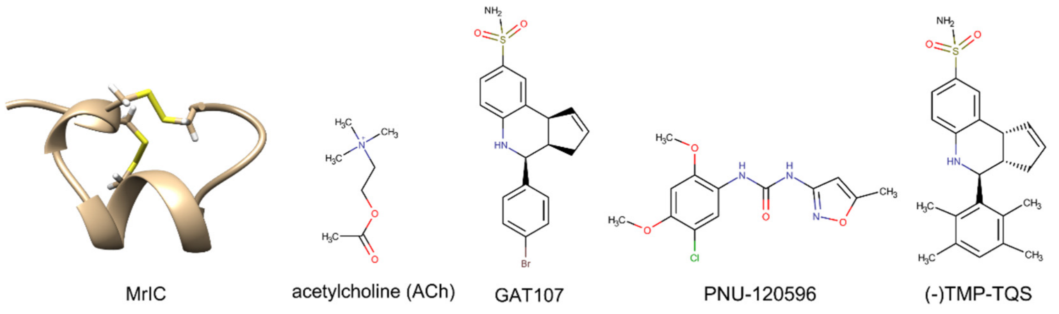

Figure S1. The structures of an MrIC model, ACh, GAT107, PNU-120596, and (-)TMP-TQS that were used in the experimental and computational studies.

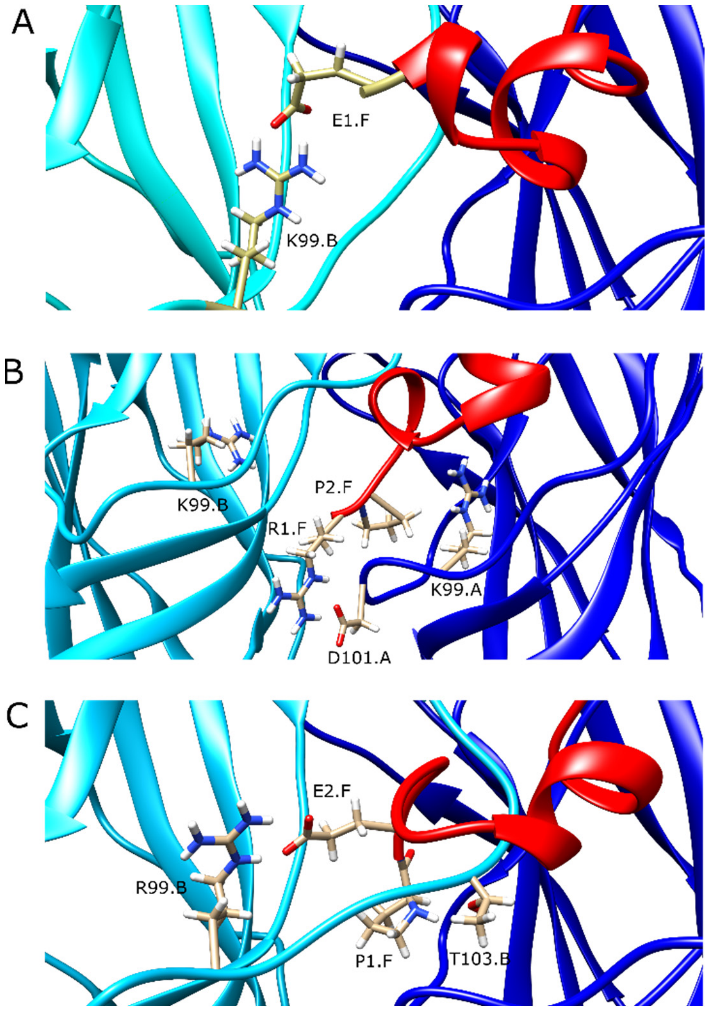

Figure S2. The interactions between MrIA (A), MrIB (B), and MrIC (C) and the vestibular residues. Chain A (blue) indicates subunit A, chain B (cyan) indicates subunit B, and chain F (red) indicates MrIC. The residues important for $\alpha 7$ nAChR – MrIC interactions are shown explicitly.

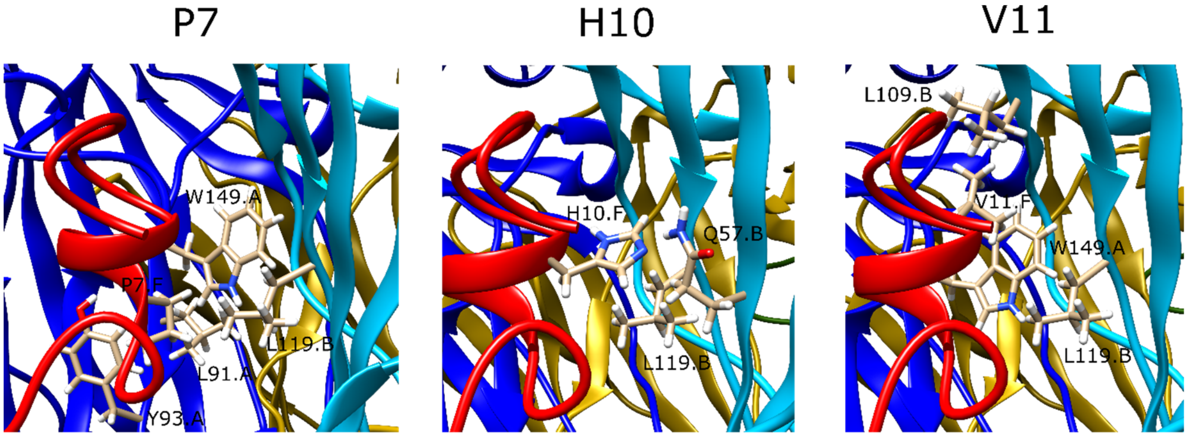

Figure S3. The lowest-scoring docking pose of MrIC at the WT $\alpha 7$ nAChR orthosteric site viewed with the important interactions that interact with P7MrIC (left), H10MrIC (middle), and V11MrIC (right) labeled. Chain A (blue) stands for the positive subunit of the interface bearing the C-loop, chain B (cyan) stands for the negative subunit of the interface, and chain F (red) stands for the peptide.

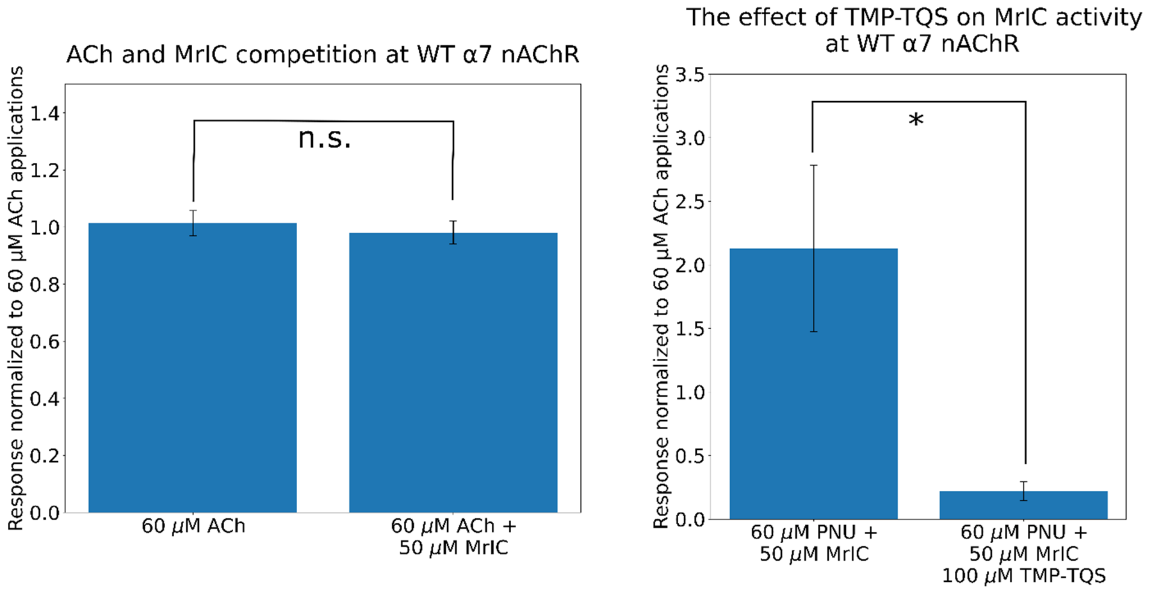

Figure S4. The effect of 50 μM MrIC application on the activity of 60 μM ACh at WT *α*7 nAChR (n=7) (A), and the effect of 100 μM (-)TMP-TQS (n=6) on the potentiated response evoked by 60 μM PNU-120596 + 50 μM MrIC (n=7) at WT *α*7 nAChR. All responses were normalized to the responses evoked by 60 μM ACh applications. n.s. stands for not significant, * stands for $p < 0.05$, ** stands for $p < 0.01$, *** stands for $p < 0.001$, and **** stands for $p < 0.0001$.

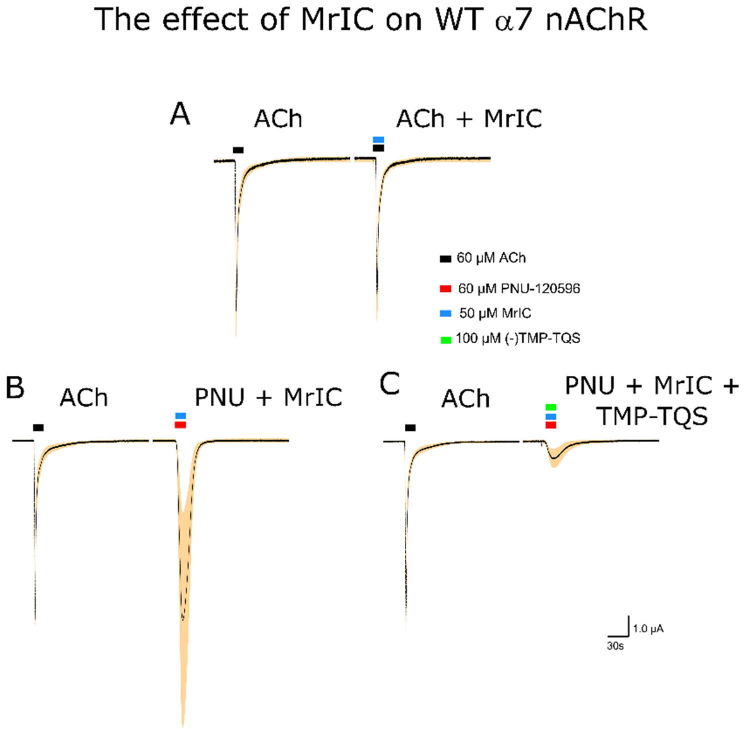

Figure S5. The current traces measured for (A) 60 μM ACh and 60 μM ACh + 50 μM MrIC applications, (B) 60 μM ACh and 60 μM PNU-120596 + 50 μM MrIC, and (C) 60 μM ACh and 60 μM PNU-120596 + 50 μM MrIC + 100 μM (-)TMP-TQS applications. The x-axis stands for the time scale of the measurement and the y-axis stands for the current induced by each application. Black bars indicate the application time of 60 μM ACh, red bars indicate the application time of 60 μM PNU-

120596, blue bars indicate the application time of 50 μM MrIC, and green bars indicate the application time of 100 μM (-)TMP-TQS. See the Materials and Methods section for a more detailed explanation on the measurements and how the error bands are calculated.

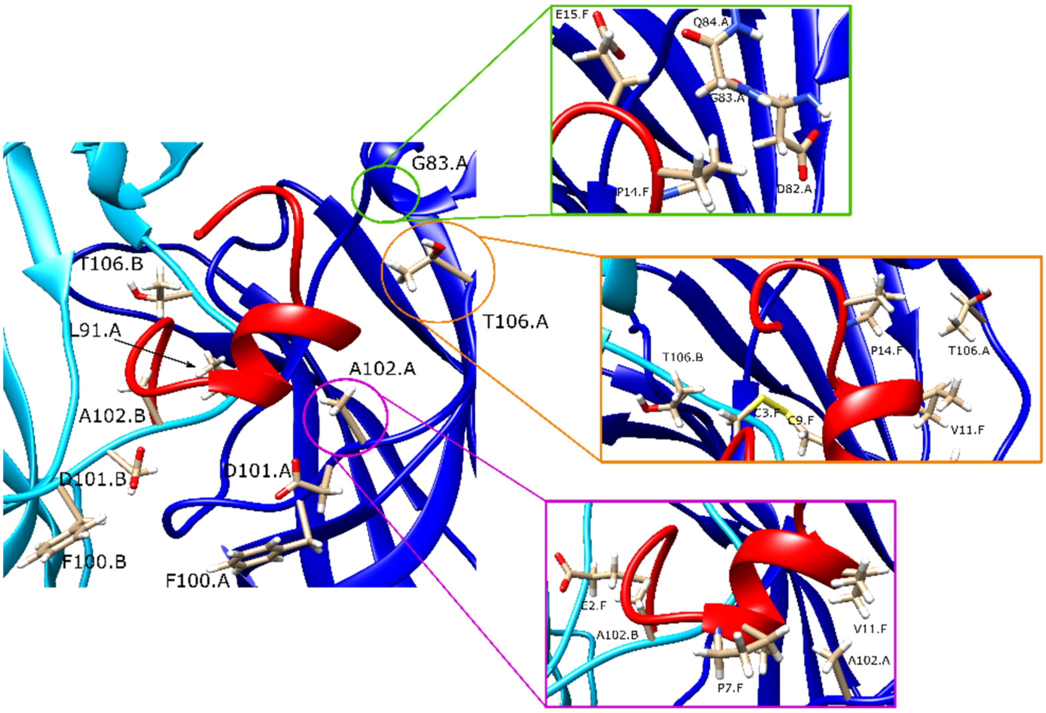

Figure S6. The lowest-scoring MrIC pose at the WT α7 nAChR AA site and the peptide-protein interactions at the vicinity of the residues G83 (green box), T106 (orange box), and A102 (purple box). The chain A (blue) indicates subunit A, the chain B (cyan) indicates subunit B, and the chain F (red) indicates MrIC. The residues important for α7 nAChR – MrIC interactions are shown explicitly.

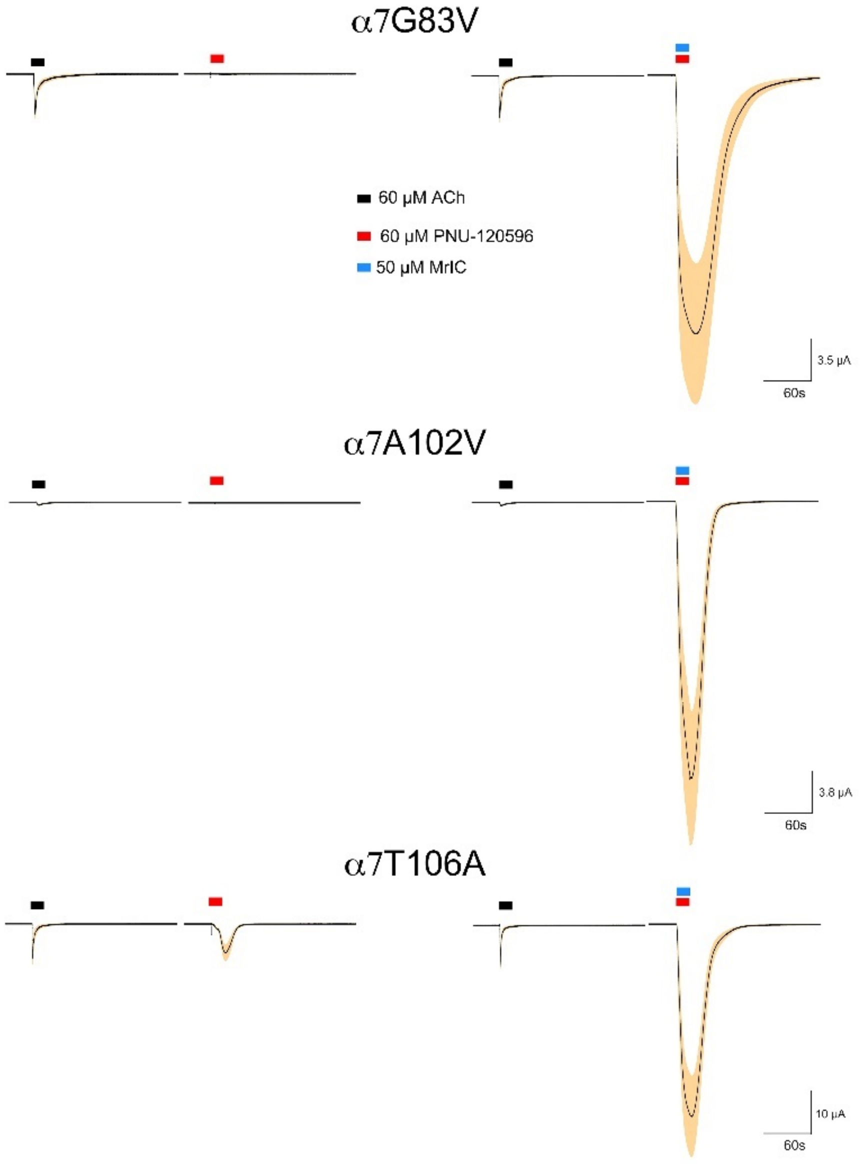

Figure S7. The current traces measured for α7G83V (top), α7A102V (middle), and α7T106A mutations. The left panels show the current induced by standalone application of 60 μM ACh (black) and 60 μM PNU-120596 (red). The right panels show the current induced by the 60 μM ACh control application (black) and the 60 μM PNU-120596 + 50 μM MrIC application. The x-axis stands for the time scale of the measurement and the y-axis stands for the current induced by each application. See the Materials and Methods section for a more detailed explanation on the measurements and how the error bands are calculated.
